# Environmental factors affecting the delivery practices of hospital-based intrapartum care

**DOI:** 10.4102/phcfm.v17i1.4615

**Published:** 2025-09-04

**Authors:** Azeh O. Eliud, Emmanuel E.-O. Agbenyeku, Teboho A. Moloi, Anesu G. Kuhudzai

**Affiliations:** 1 Department of Environmental Health, Faculty of Health Sciences, University of Johannesburg, Johannesburg, South Africa; 2 Postgraduate School, University of Johannesburg, Johannesburg, South Africa; 3 Statistical Consultation Services, University of Johannesburg, Johannesburg, South Africa

**Keywords:** childbirth, labour, maternity, quantitative research, intrapartum care

## Abstract

**Background:**

The annual World Health Organization reports confirm over 295 000 maternal deaths globally with most of these occurring during delivery. Interestingly, some studies have established a significant relationship between environmental factors and hospital-based intrapartum care.

**Aim:**

This study investigated the associated environmental factors among women presenting for peripartum care at the Ketté District Hospital.

**Setting:**

The study was conducted at the Ketté District Hospital.

**Methods:**

This quantitative cross-sectional study was conducted at the Ketté District Hospital on women presenting for peripartum care. A convenient sampling was used while a self-administered questionnaire was the data collecting tool to measure environmental factors affecting the delivery practices. Using IBM-SPSS version 29.0, logistic regression served for data analysis with statistical significance considered at *p* < 0.05.

**Results:**

The study involved 471 women presenting for peripartum care, of whom 325 (69.0%) were aged 18–25 years. Most women, 429 (91.1%), indicated having used earthed road links to the hospital. The majority agreed having suffered complications during delivery. Means of transportation (*p* = 0.010), number of past pregnancies (*p* = 0.044), place of delivery (*p* = 0.001) and delivery outcome (*p* = 0.001) were significantly associated with delivery complications.

**Conclusion:**

The study found that delivery complications were significantly associated with means of transportation to antenatal visit, place of delivery, delivery outcome and number of pregnancies.

**Contribution:**

This study contributed to a better understanding of the effects of environmental factors on the utilisation of healthcare services during the intrapartum period in rural communities of Cameroon.

## Introduction

Defined by the World Health Organization (WHO) as the death of a mother because of complications from pregnancy, up to and within 42 days following childbirth, maternal mortality (MM) has for decades remained a public health indicator of the quality of services offered by a health system.^1^ According to the 2019 WHO global factsheet, there were roughly 295 000 largely preventable deaths among women during labour, delivery and the immediate puerperal period in the 2017.^1,2^

According to global estimates, some 140 million births occur annually with the majority of these being vaginal deliveries among pregnant women. Most of these women do not present with identifiable risk factors at delivery. However, in situations where complications do arise during labour, the risk of morbidity and death becomes important for both mother and baby.^3^ The relevance of maternal and neonatal mortalities as global public health concerns is evident in the elaboration of the Millennium Development Goals (MDGs) of 2000 and later the Sustainable Development Goals (SDGs) of 2015, both of which were expounded upon by the countries worldwide during the United Nations General Assembly. Not surprisingly, target 3 of the SDG 3 aims at reducing the maternal mortality rate (MMR) to less than 70 deaths per 100 000 live births by 2030. Interventions included are the provision of evidence-based clinical and programmatic guidelines, setting global standards and providing technical support to member states on developing and implementing effective policy and programmes during pregnancy and childbirth.^1^

Almost 94% of the approximately 295 000 preventable maternal deaths that occurred in 2017 were in low-resource settings. Disturbingly, 86% of these deaths occurred in sub-Saharan Africa and Southern East Asia alone.^2^ It is worth noting that one-third of maternal deaths and pregnancy-related morbidities are accounted for by complications arising during labour, childbirth and the immediate postpartum period. Most of these deaths could largely be prevented if access to the health facility were readily feasible, pregnancies and deliveries were attended to by skilled health personnel having the right equipment and supplies and complicated cases were timely referred to equipped emergency obstetric units.^1,4^

Despite global efforts, the situation remains deplorable in most developing lands. In Cameroon, WHO reported an alarming 529 MMR (per 100 000 live births) in 2016 while the neonatal mortality rate (NMR) was estimated at 24 deaths per 1000 live births.^5^ Not only does this place the country off target as far as attaining the ambitious SDGs, but it also puts the country well above global and regional values.^6,7^ As a result, local policies have involved training health personnel, implementing the systematic utilisation of the partogram to follow up all deliveries and the implementation of regular surveillance meetings at district and regional levels to identify and redress any causes of evitable MM or morbidity. Nonetheless, with more equipped health services concentrated in major cities, women living in remote areas find it especially difficult to access emergency obstetric care for childbirth. This is compounded by the long travel distances, limited transport options, the absence of skilled staff and adequate drugs or equipment, all of which make hospital-based delivery services nearly as unsafe as deliveries carried out by unskilled traditional birth attendants in the community.^8^

Interestingly, several studies have evoked the impact of environmental and geographic factors as major determinants to the accessibility to healthcare delivery and hence the health outcomes.^9,10,11,12,13,14,15^ With a persistent gap in knowledge on the perceived impact of environmental and geographic factors on maternal and early neonatal healthcare services offered in most health facilities, there is an urgent need to understand the effect of these on the health status of these vulnerable groups and how and why certain strategies should be implemented. This should help rationalise programming decisions.^5,7,16^ This is in line with a quantitative cross-sectional survey in Ghana where suboptimal access and utilisation of healthcare services during the maternal period was found to be influenced by the socio-economic characteristics of pregnant women.^7^ Therefore, it was considered that the results of this study will be beneficial in finetuning efforts aimed at improving upon healthcare utilisation by this vulnerable group in a bid to achieve global targets.

As outlined by the Health Belief Model (HBM),^13^ a woman’s perception on intrapartum care is critical to her health-seeking behaviour. The perceived distance in reaching the healthcare service, the estimated cost of care and perceptions on the quality of care affect decision making (first delay). Additionally, the second delay, that of reaching the health facility, is related to the distribution and site of health facilities capable of offering optimal maternal and neonatal care, as well as the availability and accessibility of transportation means to the health facilities.^1,10,11,17,18,19,20^

In Cameroon where these factors weigh on the nation’s health system, very little has been done to identify and address the situation. This could partly be because of the absence of data to concretise knowledge on these gaps, resulting in limited policy inclusion. This hinders progress toward sustainable development and universal health coverage. It is in this light that the current study aimed to assess the effects of environmental factors, delivery outcomes and other characteristics such as geographical factors on the utilisation of hospital-based intrapartum care. This unique study conducted in the Ketté Health District will address the absence of substantial information on understanding the difficulties associated with the accessibility to healthcare services. In 2019, Cameroon began implementing the decentralisation process with the involvement of local communities and councils in service delivery. Hence, findings from this study will inform the decisions and implementation of developmental strategies through the country’s decentralisation plan to offer quality healthcare to its population.

## Materials and methods

This was a quantitative cross-sectional, hospital-based survey, which was carried out at the Ketté District Hospital, a rural health district located in the East region of Cameroon. The locality is found at an altitude of 764 m above sea-level, having a savannah-type vegetation and a mixed population of 65 550 inhabitants, principally composed of the local ‘Gbaya’ community, the Fulani and an important refugee population from neighbouring Central African Republic.^6^ Roads linking health facilities within the district health area are earth roads, being barely accessible during the rainy season.

The study population included women of reproductive age, between 15 and 49 years, residing within the Ketté Health District area. For ethical reasons and in line with national standards, eligible participants were pregnant women aged between 18 and 49 years inclusive, who presented at term (i.e., at least completed 36 weeks of amenorrhoea) for antenatal consultation, for intrapartum or for early postpartum care (within the first 2 weeks following delivery, regardless of the delivery outcome) at the Ketté District Hospital and who voluntarily consented to participation. Persons who objected to participate or who did not meet the specific inclusion criteria were excluded from the study.

The G*power software (version 3.1.9.7) was used to do an *a priori* power analysis,^21^ and the findings showed that the sample size was adequate for valid results to identify small, medium and large effects. With a critical T value of 1.96 and a power of 0.8, alpha level of 0.05 and low effective size of 0.2, we were able to get the necessary sample size of 471 people.

The convenience sampling method was used. For this, an institution-based cross-sectional study at the Ketté District Hospital was conducted. To compensate for the passive exclusion of women not presenting at the retained health facility for peripartum care, all patients presenting at the waiting room of the hospital were notified by the data collectors of the ongoing research, its purpose, objectives, admission and exclusion criteria, the methodology and the potential benefits to the population. This was done as a public announcement. All eligible and willing participants were then given the opportunity to provide written consent at the end of their regular visit. Where necessary, questions were rephrased into the local language to ease understanding.

A structured closed-ended, self-administered questionnaire developed from literature review and published studies was the tool used for data collection. A pilot study was conducted to test the quality of the questionnaire and to ensure the internal integrity of the tool.^12,22^ Data were then collected as primary data and were grouped based on environmental, socio-demographic, and obstetric and delivery characteristics. These were further categorised as exposure variables, outcome variable and covariates or confounders. Where applicable, a Likert scale was used to gauge opinions and attitudes.^9,22,23^

Data were prepared and analysed using IBM Corp. (Released 2023, IBM SPSS Statistics for Windows, Version 29.0.2.0 Armonk, NY: IBM Corp statistics version 29.0).^24^ Descriptive statistic such as frequencies and percentages were computed to summarise the nominal and ordinal variables, while means and standard deviations (s.d.) were used to describe the numerical variables. The Chi-square test was computed to evaluate the association between categorical determinants and the outcome variable(s). Logistic regression analysis was performed to identify significant predictors of suffering complications during delivery. A *p* < 0.05 was considered statistically significant.

The reliability of the data collection tool was ensured through a test-retest analysis, which was performed on 5% of the sample group. External validity was ensured by raising the sample size to a higher level as determined by the time frame. For internal validity, questions were proofread, translated into the local language and re-read by the supervisor and three different translators to ensure the use of simple understandable language, the absence of jargon or double-barrel questions.

For ethical considerations, the study proposal was submitted to and approved by the University of Johannesburg’s Higher Degree Committee (HDC) and the Research Ethics Committee (REC) while written authorisation was obtained from the Regional Delegate of Public Health for the East Region. The use of face masks, hand sanitisers, physical distancing of at least one meter between the interviewer and the participant was compulsory throughout the data collection process to ensure the application of standard barrier measures within the context of the coronavirus disease 2019 (COVID-19) pandemic.

### Ethical considerations

Ethical approval to conduct this study was obtained from the University of Johannesburg Faculty of Health Sciences Higher Degree Committee MPHHDC-01-07-2021.

## Results

The study’s data and findings are shown in this section. IBM-SPSS version 29.0^24^ was used for data preparation and analysis. Data include respondents’ delivery and obstetric details, as well as environmental elements and demographics. Along with submitting the analysis, it talks about the outcomes by making predictions and looking for correlations.

### Demographic information

The number and percentage of participants’ biographical details are shown in Table 1. A total of 471 participants were included in this study, with the majority being married 325 (69.0%). The most common age group was 18–25 years (*n* = 207, 43.9%). A sizeable portion of the participants had more than six members that make up their family (*n* = 146, 31.0%). The majority of participants were Muslim (*n* = 255, 54.1%), with 301 (63.9%) having a primary education and 349 (74.1%) were unemployed.

**TABLE 1:** Biographical details of study participants.

Characteristics	Category	*n*	%
**Demographic data**			
Age (years)	18–25	207	43.9
	26–33	144	30.6
	34–41	88	18.7
	42–49	32	6.8
Marital status	Single	125	26.5
	Married	325	69.0
	Widowed	21	4.5
Family size	1–2	58	12.3
	3–4	146	31.0
	5–6	121	25.7
	More than 6	146	31.0
Religion	Christian	211	44.8
	Muslim	255	54.1
	Atheist	5	1.1
Level of education	Primary	301	63.9
	Secondary	78	16.6
	Tertiary	10	2.1
	Other	30	6.4
	Illiterate	52	11.0
Employment status	Unemployed	349	74.1
	Employed	43	9.1
	Self-employed	59	12.5
	Other	20	4.2
**Environmental and geographical characteristics**			
Means of transportation used to get to the hospital	Vehicle or Motorcycle	214	45.4
	Foot	257	54.6
Type of road linking home to hospital	Earthed	429	91.1
	Footpath	42	8.9
Season of the year you last gave birth	Rainy season	257	54.6
	Dry season	214	45.4
**Obstetric and delivery information**			
Suffered from any complications during delivery	Yes	114	24.2
	No	357	75.8
Baby suffered from any complications following delivery	Yes	132	28.1
	No	339	71.9

The majority of the participants (*n* = 257, 54.6%) walked to the hospital, while 429 (91.1%) travelled an earthed route and 257 (54.6%) had their babies during the rainy season. Furthermore, the majority of the 357 participants (75.8%) reported problems during their delivery. However, majority (*n* = 339, 71.9%) reported that their infant did not suffer any complications after delivery.

### Perceived importance, influence and satisfactory environmental factors

Table 2 to Table 4 show the frequency count, percentage responses, means and s.d. for each of the items measuring antenatal care (ANC) visit, place of delivery, the delivery outcome, satisfactory ANC frequencies and delivery outcome. The responses were measured using 5-point Likert scale. However, for ease of interpretation, the two lower and two upper scales were combined.^25^

**TABLE 2:** Perceived importance of environmental factors during antenatal care visit.

ACV items	Unimportant and slightly unimportant		Moderately important		Important and very important		Mean	s.d.
	*n*	%	*n*	%	*n*	%		
ACV1. Quality of the road	141	29.9	63	13.4	267	56.7	3.12	1.358
ACV2. Time from home	131	28.5	71	15.1	266	56.4	3.22	1.360
ACV3. Distance from home	117	24.8	77	16.3	277	58.8	3.32	1.352
ACV4. Means of transportation	139	29.5	75	15.9	257	54.6	3.17	1.405
ACV5. Season of the year	170	36.1	63	13.4	238	50.5	3.00	1.438
ACV	-	-	-	-	-	-	3.166	1.382

s.d., standard deviations; ACV, antenatal care visit.

**TABLE 3:** Place of delivery and the delivery outcome.

PD Items	Not and slightly influential		Moderately influential		Influential and completely influential		Mean	s.d.
	*n*	%	*n*	%	*n*	%		
PD1. Quality of the road	206	43.7	71	15.1	194	41.2	2.67	1.396
PD2. Time from home	156	33.1	60	12.7	255	54.2	3.06	1.378
PD3. Distance from home	155	33.0	56	11.9	260	55.2	3.07	1.387
PD4. Means of transportation	189	40.2	55	11.7	227	48.2	2.91	1.478
PD5. Season of the year	255	54.2	67	14.2	149	31.7	2.40	1.419
PD	-	-	-	-	-	-	2.822	1.412
**DO Items**								
DO1. Quality of the road	199	42.2	91	19.3	181	38.4	2.70	1.347
DO2. Time from home	140	29.7	81	17.2	250	53.1	3.14	1.379
DO3. Distance from home	135	28.7	94	20.0	242	51.3	3.16	1.385
DO4. Means of transportation	180	38.3	84	17.8	207	40.0	2.94	1.403
DO5. Season of the year	258	54.8	78	16.6	135	28.6	2.35	1.386
DO	-	-	-	-	-	-	2.859	1.380

s.d., standard deviations; PD, place of delivery; DO, delivery outcome.

**TABLE 4:** Satisfactory antenatal care frequencies and satisfactory delivery outcome.

SANCf items	Not at all and slightly satisfied		Moderately satisfied		Very and completely satisfied		Mean	s.d.
	*n*	%	*n*	%	*n*	%		
SANCf1. Quality of the road	224	47.6	64	13.6	183	38.9	2.620	1.402
SANCf2. Time from Home	179	38.0	58	12.3	234	49.7	2.940	1.456
SANCf3. Distance from home	161	34.1	74	15.7	236	50.1	3.040	1.405
SANCf4. Means of Transportation	175	37.2	72	15.3	224	47.6	2.990	1.462
SANCf5. Season of the year	245	52.0	66	14.0	160	33.9	2.460	1.416
SANCf	-	-	-	-	-	-	2.809	1.428
**SDO Items**								
SDO1. Quality of the road	251	53.3	167	35.5	53	11.2	2.330	0.989
SDO2. Time from Home	241	51.2	161	34.2	69	14.6	2.460	0.969
SDO3. Distance from home	225	47.7	169	35.9	77	16.3	2.530	0.954
SDO4. Means of transportation	277	58.8	128	27.2	66	14.0	2.360	1.026
SDO5. Season of the year	305	64.7	109	23.1	57	12.1	2.200	0.998
SDO	-	-	-	-	-	-	2.378	0.987

s.d., standard deviations; SANCf, satisfactory antenatal care frequencies; SDO, satisfactory delivery outcome.

Analysis reported in Table 2 demonstrates that all items had a more positive response towards patient’s ANC visits, as seen by the mean scores that are above 3. Furthermore, 277 (58.8%) participants indicated that the distance from home is important/very important when attending antenatal visits at the health facility, and 267 (56.7%) corroborated that it is important/very important to have a quality road when attending the antenatal visits. Additionally, 266 (56.4%) participants indicated that time is important/very important when attending antenatal visits, and 257 (54.6%) indicated that means of transportation is important/very important when attending antenatal visits. As evident in Table 2, a total of 238 participants (50.5%) saw the importance of the season of the year when attending antenatal visits. Finally, the summated mean score of 3.166 and s.d. of 1.381 indicate an overall importance of the items measuring the antenatal care visit (ACV) construct.

The analysis in Table 3 shows that 2 out of 5 mean score values are greater than 3, suggesting how crucial the elements evaluating the place of delivery are. In all, 260 (55.2%) participants stated that distance from home is influential/completely influential when it comes to place of delivery, whereas 255 (54.2%) responded that time is crucial/completely influential. Table 3 further indicates that 3 of the 5 items had no influence on place of delivery evidenced by the mean values below 3. For 227 (48.2%) participants, mean of transportation to place of delivery is not at all/slightly influential, while a total of 206 participants (43.7%) indicated that the quality of the road to the place of delivery is not at all/slightly influential. Furthermore, 255 (54.2.7%) participants stipulated that the season of the year is not at all/slightly influential regarding place of delivery. Finally, the summated mean score of 2.822 and s.d. of 1.412 indicate an overall no influence on the items measuring the place of delivery (PD) construct.

Table 3 also indicates that 2 of the 5 mean score values are higher than 3, indicating the influence of the items assessing the delivery outcomes. According to 250 (53.1%) participants, time from home is influential/completely influential regarding delivery outcome, while distance from home is influential/completely influential according to 242 (51.3%) participants. Table 3 furthermore indicates that 3 of the 5 items had no influence on the place of delivery as evidenced by the mean values below 3. Consequently, 258 (54.8%), 207 (42.2%) and 207 (40.0%) participants, respectively, indicated that season of the year, quality of the road and means of transportation have no influence at all or slight influence regarding the delivery outcome. Finally, the summated mean score of 2.859 and s.d. of 1.380 indicate an overall importance to the items measuring the delivery outcome (DO) construct.

It is evident from Table 4 that only one mean score value is above 3, indicating high level of satisfaction with the item (distance form home) used to measure the satisfaction of antenatal care frequencies (SANCf) of the antenatal visits construct. In all, 236 (50.1%) participants were very/completely satisfied with the distance from home regarding the ANC frequencies of the antenatal visits. While 245 (52.0%), 234 (49.7%) and 224 (47.6%)participants, respectively, were not at all/slightly satisfied with the items season of the year, time from home, means of transportation and quality of the road used to measure the ANC frequencies of the antenatal visits. In conclusion, the total mean score of 2.809 and the s.d. of 1.428 suggest a general discontentment with the questions that assess the SANCf construct.

Furthermore, it is discernible from Table 4 that all the mean score values are below 3, indicating a high level of dissatisfaction with the items used to measure the delivery outcome (DO) construct. A total of 205 (64.7%) participants stipulated that they were not at all/slightly satisfied with the season of the year, 277 (58.8%) were not at all/slightly satisfied with the means of the transportation, 251 (53.3%) were also not at all/slightly satisfied with the distance from home and 241 (51.2%) participants were also not at all/slightly satisfied with the time from home regarding the items measuring the DO construct. To sum up, an overall lack of satisfaction with the items measuring the satisfaction of DO (SDO) construct is shown by the summated mean score of 2.378 and s.d. of 0.987.

### Validity and reliability

Table 5 shows the outcomes of internal consistency and explores the structure of ACV, PD, DO, SANCf and SDO constructs; the items were subjected to an exploratory factor analysis (EFA) with principal axis factorising. It is evident that each construct factored out to a single factor using Kaiser’s eigenvalue of greater than one criterion.^26^ The variance explained by all the five factors from each construct is above 60%, which is consistent with earlier studies.^27,28^ Moreover, Table 5 demonstrates that every construct’s factor loading is over the 0.5 threshold, demonstrating its paramount importance to the components it loads.^29^

**TABLE 5:** Exploratory factor analysis and reliability of the measurement scale.

Constructs	Factor loadings	Eigenvalues	Variance explained by (%)	Kaiser-Meyer-Olkin	Bartlett’s test of sphericity	Cronbach’s alpha
ACV items	-	3.597	66.085	0.806	< 0.001	0.898
ACV1	0.934					
ACV2	0.893					
ACV3	0.816					
ACV4	0.804					
ACV5	0.566					
PD items	-	3.824	71.180	0.824	<0.001	0.921
PD1	0.917					
PD2	0.894					
PD3	0.867					
PD4	0.849					
PD5	0.667					
DO items	-	3.646	67.433	0.815	< 0.001	0.904
DO1	0.932					
DO2	0.921					
DO3	0.844					
DO4	0.795					
DO5	0.559					
SANCf items	-	3.648	67.385	0.824	<0.001	0.904
SANCf1	0.925					
SANCf2	0.896					
SANCf3	0.882					
SANCf4	0.779					
SANCf5	0.572					
SDO items	-	3.685	67.755	0.858	< 0.001	0.909
SDO1	0.911					
SDO2	0.885					
SDO3	0.849					
SDO4	0.792					
SDO5	0.653					

PD, place of delivery; DO, delivery outcome; SANCf, satisfactory antenatal care frequencies; SDO, satisfactory delivery outcome; ACV, antenatal care visit.

The measure of sampling adequacy (MSA) of factor analytic data matrices was first introduced by.^26^ Then,^30^ modified it. The Kaiser-Meyer Olkin (KMO) measurement of sampling adequacy of 0.806 for the ACV construct, 0.824 for the PD construct and 0.815 for the DO construct in Table 5 clearly shows that the data set was appropriate for factor analysis. Furthermore, the good KMO value of 0.824 received for the construct SANCf and 0.858 obtained for the SDO construct^31,32^ suggested that the KMO > 0.8 is meritorious. In addition to validating the results of the KMO measure of sample adequacy, the Bartlett’s test of sphericity showed a significant *p* = 0.000 < 0.01 level of significance.^33^ This suggests that the items had a strong or moderate association.

The dependability of the instrument was assessed using an internal consistency analysis. The ability of an instrument to produce consistent results over time is known as reliability.^34^ The findings of the reliability analysis performed on the five extracted elements revealed that the Cronbach’s alpha coefficients for the ACV, PD, DO, SANCf and SDO were 0.898, 0.921, 0.904 and 0.9092, respectively. Since all Cronbach’s alpha coefficient values are much greater than the minimum allowed value of 0.7,^35,36^ the scale is considered reliable.

### The association between delivery complications and demographics data, antenatal visit, obstetric history and outcomes

To compare experiencing delivery problems with ANC visits, a Chi-square test was employed. Based on the findings, the only item that showed a significant correlation with experiencing delivery problems was the mode of transportation (*p* = 0.010). Subsequently, a significant correlation was found (*p* = 0.044) between the number of pregnancies held by the participants and the problems suffered during delivery. This association also extended to the obstetric history, outcomes and suffering complications during delivery. Table 6 indicates a substantial correlation between experiencing problems during delivery and the location of the birth (*p* = 0.001) and the pregnancy’s outcome (*p* = 0.001). These results are consistent with those of the earlier studies.^37^

**TABLE 6:** Association between delivery complications and demographics data, antenatal visit, obstetric history and outcomes.

Characteristics	Categories	Did you suffer from any complications during delivery?		*p*
		Yes	No	
**Demographic data**				
Which age group best describes you?	18–25 years	40	138	0.124
	26–33 years	45	94	
	34 years or more	29	89	
What is your marital status?	Not married	41	95	0.127
	Married	73	226	
How many persons make up your family?	1–4 members	41	133	0.181
	More than 5 members	71	188	
What is your religious affiliation?	Christian and Atheist	50	155	0.241
	Muslim	64	166	
What is your level of education?	Primary or below	80	209	0.193
	Secondary and above	34	112	
What is your employment status?	Unemployed	90	231	0.090
	Employed	24	90	
**Antenatal Care visit**				
	Unimportant and Slightly Important	35	88	
ACV1. Quality of the road	Moderately Important	17	44	0.704
	Important and Completely Important	62	189	
	Unimportant and Slightly Important	27	90	
ACV2. Time from home	Moderately Important	20	48	0.607
	Important and Completely Important	67	183	
	Unimportant and Slightly Important	27	75	
ACV3. Distance from home	Moderately Important	24	49	0.332
	Important and Completely Important	63	197	
	Unimportant and Slightly Important	44	79	
ACV4. Means of transportation	Moderately Important	18	47	0.010
	Important and Completely Important	52	195	
	Unimportant and Slightly Important	31	121	
ACV5. Season of the year	Moderately Important	22	39	0.052
	Important and Completely Important	61	161	
**Obstetric history and outcomes**				
How many times have you been pregnant?	Two times or less	40	144	0.044
	More than two times	74	177	
Where did your last delivery take place?	Hospital and Health Centre	34	151	<0.001
	Traditional birth attendant and other	80	170	
What was the outcome of the pregnancy?	Healthy baby	42	275	<0.001
	Unhealthy and Dead baby	72	42	

ACV, antenatal care visit.

### The logistic regression analysis of the association between obstetric history and outcomes, environmental factors and satisfactory

Table 7 showed that participants who delivered using a traditional attendant or other had 0.347 higher odds of suffering complications during delivery compared to those who delivered at the hospital/health centre (*p* = 0.004). Similarly, participants who delivered unhealthy baby had 0.089 times higher odds than those who delivered a healthy baby (*p* = 0.001) (Table 7). Furthermore, the results indicate that the increasing PD, SANCf and SDO are associated with an increased likelihood of suffering from complications during delivery.

**TABLE 7:** Logistic regression analysis of the association between obstetric history and outcomes, environmental factors and satisfactory.

Did you suffer from any complications during delivery? (yes)	Adjusted odds ratio	95% confidence interval		*p*
		Lower bound	Upper bound	
What means of transportation do you mostly use to get to the hospital?	0.841	0.456	1.551	0.579
What type of road links your home to the hospital?	3.024	0.764	11.966	0.115
During which season of the year did you last put to birth?	0.714	0.413	1.234	0.228
ACV	1.159	0.863	1.556	0.327
How many times have you been pregnant?	0.612	0.354	1.060	0.080
Where did your last delivery take place?	0.347	0.167	0.720	0.004
What was the outcome of the pregnancy?	0.089	0.051	0.154	<0.001
PD	0.487	0.272	0.871	0.015
DO	0.948	0.544	1.593	0.841
SANCf	1.6869	1.175	2.426	0.005
SDO	0.694	0.486	0.991	0.044

PD, place of delivery; DO, delivery outcome; SANCf, satisfactory antenatal care frequencies; SDO, satisfactory delivery outcome; ACV, antenatal care visit.

## Discussion

This study articulated the effect of environmental factors on hospital-based intrapartum care tendencies in a rural setting of Cameroon with findings being similar to several other studies.^15,38,39,40^ As regard to socio-demographic indicators, participants with a family size of 1–2 persons were less likely to have had maternal complications, a finding concordant with those obtained earlier.^41^ This seems logical as the financial capacity to afford for healthcare services is diluted by an increase in family size, a predisposition to intrapartum complications as opposed to a lower likelihood for maternal complications when the family size is smaller.^42^ Not surprisingly, unemployed participants were more likely to have had maternal complications, as was the case in other studies.^15,43,44^

Women above 35 years showed more responsible behaviour and were more confident and influential in decision-making as opposed to younger women, resulting in an increased utilisation of ANC services and lesser odds for maternal complications. Similar observations from other studies revealed that single mothers were more likely to have had maternal complications, possibly because of the increased exposure to lesser financial support.^10,39,41^ As with some studies, no statistically significant association was identified between religious affiliation and the likelihood for delivery complications.^10^ Consistent with findings made by other researchers was the relationship between a limited level of education and maternal complications.^10,40,42,45,46^ This suggests that increased maternal education has a positive effect on complication preparedness, hence reducing the risk of maternal complications.^39,41,47,48^

No significant relationship was found in this study between gestational age and delivery complications. This contradicts results from other studies where an increase in gestational age after 40 weeks was strongly associated with greater odds for maternal complications because of placental insufficiency, macrosomia, meconial aspiration and decreased amniotic fluid.^42,49^ In this study, only 31 participants (6.6%) had attended at least 4 ANC visits as opposed to the required minimum of 8 ANC visits. These findings correlate with an earlier study^15^ in which only 9.1% of participants had attended at least 4 visits with a significant correlation between number of ANC and delivery complications. It has been established that the lesser the number of ANC attended, the greater the likelihood for maternal complications because of a lower likelihood for an early detection and management of evitable risks and complications related to delivery.^9,39,45^

Participants who had been pregnant just once or thrice were less likely to have had maternal complications. This is consistent with previous findings^10,45,46^ as an increase in parity (4 or more pregnancies) has been found to have a significantly negative effect on the health-seeking behaviour during the intrapartum period as well as a higher tendency for maternal complications. Also, multiparous women (4 or more pregnancies) were more likely to rely on experience rather than seek medical care with a resultant increase in the likelihood for maternal complications.^10,45^ Participants whose deliveries were assisted by traditional birth attendants were more likely to have had maternal complications with as much as 237 (50.7%) participants having had their deliveries assisted by traditional birth attendants (TBAs). The finding from this study concords with results obtained in other studies.^9,15^ This is plausible as an increased familiarity with a TBA – if the last delivery went well – lowers the tendency to opt for medicalised care, resulting in a higher predisposition for maternal complications.^10^ More so, participants whose babies suffered from neonatal complications were more likely to have had maternal complications. This finding also concurs with results obtained from earlier studies.^49,50,51^ The occurrence of complications such as gestational diabetes, macrosomia, malformations, intrauterine growth retardation and still births have been shown to increase the risk for other intrapartum complications.^50,52,53^

## Conclusion

In this study, a strong relationship between maternal outcomes and socio-demographic and environmental determinants as determined by the use of hospital-based intrapartum health services in a rural community of Cameroon was established. This study showed that a smaller family size, walkable distance to the health facility or a low number of deliveries were predictive for poor maternal outcomes with a strong correlation between the means of transportation used, travel distance or time, season or road quality at the time of delivery and maternal outcomes. Consequently, an increased likelihood for maternal complications was found when environmental factors were not viewed as satisfactory. These findings corroborate with those observed in several other studies and highlight the need for an improved accessibility to health facilities in a bid to promote their usage, thus reducing the risks for maternal and neonatal complications especially during the intrapartum period. It is therefore essential that cost-effective and sustainable measures such as regular road maintenance, especially of secondary and tertiary roads, and the provision of motorised means of transportation to ease medical interventions be envisaged, especially in rural communities. Also, the provision of referral advice to women of child-bearing age and the training of community health workers and TBAs on first aid skills, danger signs identification and management, recommended health practices and referral could greatly improve maternal outcomes.
